# Acral Hypomelanocytic Melanoma of Left Great Toe: A Rare Cancer

**Published:** 2017-09

**Authors:** Mohd Altaf Mir, Varun Chauhan, Ali Adil Mahmud, Lalit Mohan Bariar, Suhailur Rehman

**Affiliations:** Department of Plastic and Reconstructive Surgery, Jawaharlal Nehru Medical College, Aligarh Muslim University, Aligarh, India

**Keywords:** Acral, Malignant melanoma, Hypomelanocytic

**DEAR EDITOR**

The Malignant melanoma is an uncommon primary malignant tumour of the foot.^1^ It occurs in different forms like superficial spreading, nodular, lentiginous and acral. Amelanotic/hypomelanotic melanoma is a non-pigmented variety of cutaneous melanoma accounting for 2-8% of all primary melanomas.^2^^,^^3^ The lesion is usually exophytic and ulcerative with or without metastasis. The lesions usually begin in subungal location of toe and because of absence of pigment, the lesion is often misdiagnosed. Hence diagnosis by histopathological examination and metastatic work up is necessary.^2^^,^^3^ We highlight a case of primary amelanotic melanoma of the left great toe treated by digital amputation.

A 65-year-old male presented with a 10 month old painful ulcero-proliferative growth on the great toe of the left foot. The small nodular lesion was started on plantar aspect of the toe and progressed to the size of 4x5 cm with development of ulceration and irregular margins over the period of 3 months (Figure 1). On clinical examination no lymphadenopathy was palpable. The digital amputation of left great toe with 1 cm margin clearance (Figure 2) was done and specimen sent for histopathological examination. Histopathological microphotograph (Figure 3) shows nodular aggregates of course pigmented malignant cells extending deeply within the dermis with abnormal mitosis suggestive of malignant melanoma.

**Fig. 1:**
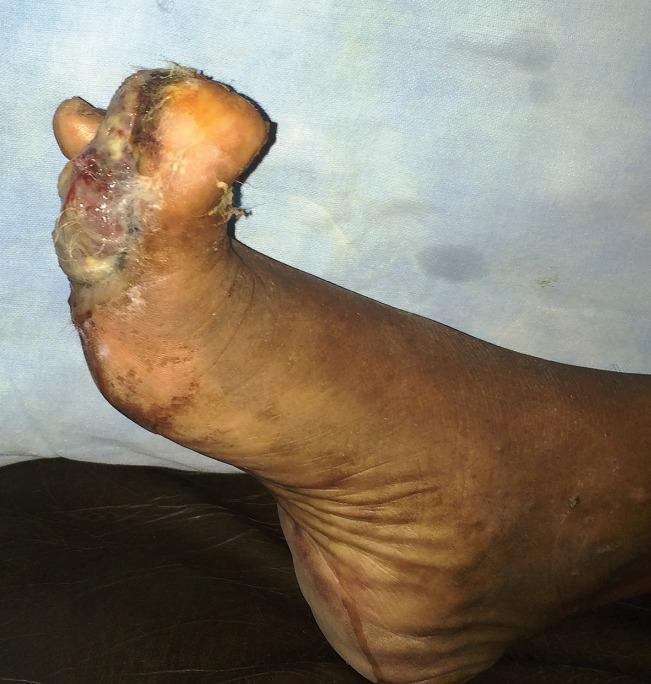
Ulceroproliferative lesion of the left great toe

**Fig. 2:**
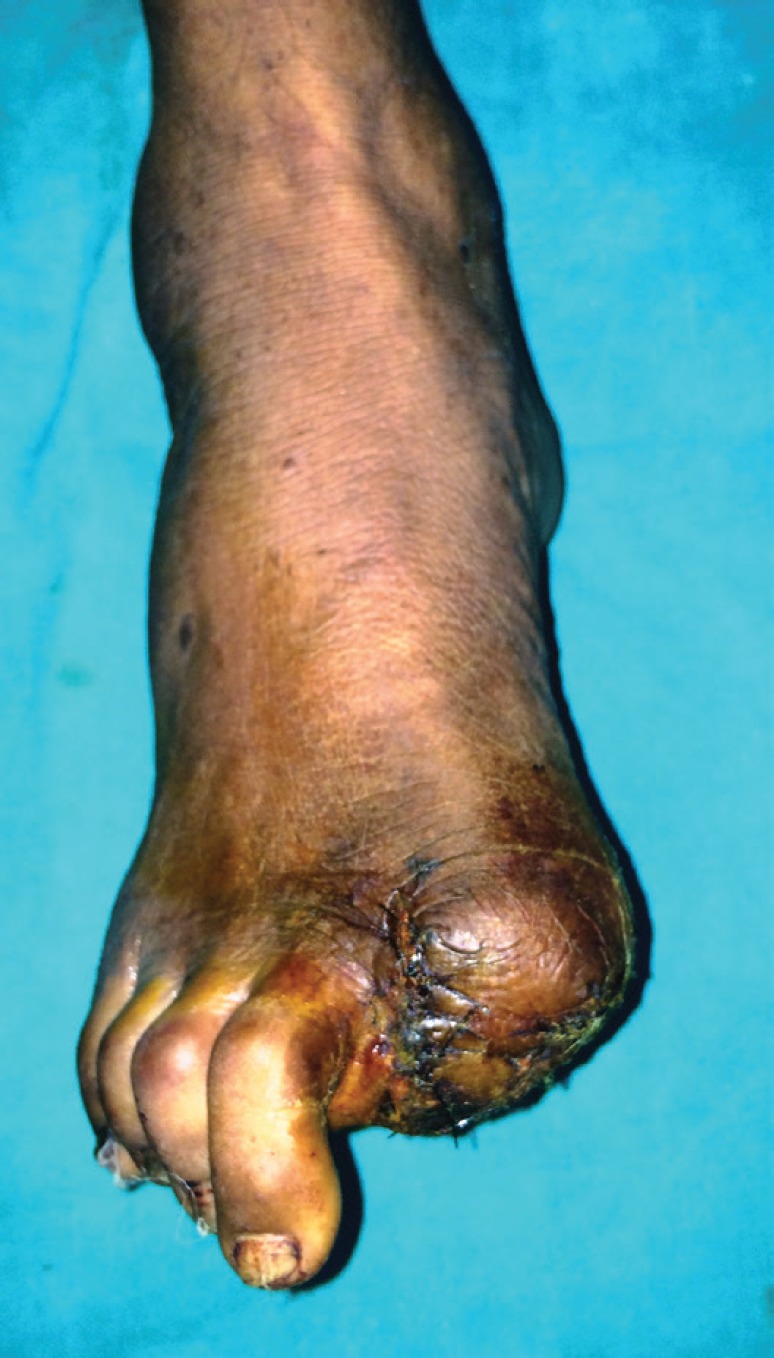
Postoperative photograph after digital amputation

**Fig. 3:**
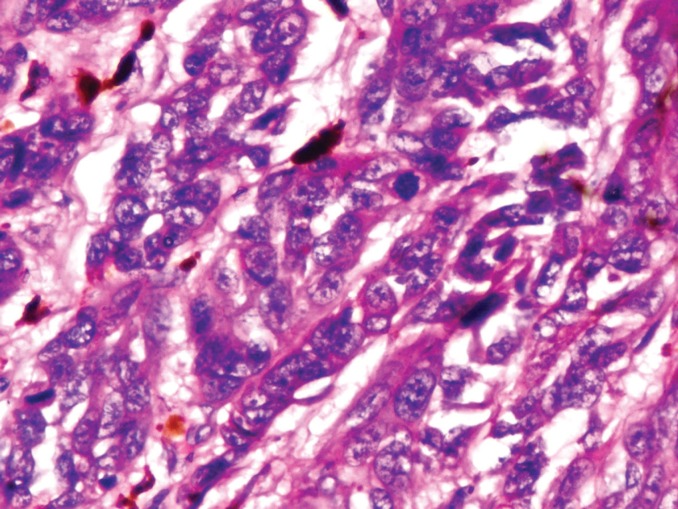
Histology of hypomenocytic melanoma

Amelanotic/hypomelanocytic malignant melanoma most often occur in sun-exposed skin of elderly people.^4^ These melanomas comprise only 2-8%^2^^,^^3^ of melanomas and clinical features mimic a variety of benign and malignant skin conditions and therefore are commonly misdiagnosed. Early diagnosis is vital for the effective management of this condition. Amelanotic/hypomelanocytic melanoma is most commonly subungual, localized and appearing like an exophytic papular or plaque-like reddish lesion and is often ulcerated.^4^ Despite the lack of pigmentation of these lesions special stains and immunohistochemistry will confirm the melanocytic nature of the lesion.^5^^-^^8^ Common clinical misdiagnoses of amelanotic melanoma include basal cell carcinoma, seborrhoeic keratosis, pyogenic granuloma, naevus, keratoacanthoma, verruca vulgaris, dermatitis, actinic keratosis, dermatofibroma and Bowen’s disease.^4^ The misdiagnosis rates of 33–67% have been reported in literature.^9^^,^^10^ In summary, though amelanotic melanoma of the toe is a rare clinicopathological entity in foot engender a life and demand early biopsy for confirmation of diagnosis with histopathology. 

## CONFLICT OF INTEREST

The authors declare no conflict of interest.
